# Onlay Technique in Incisional Hernia Repair—A Systematic Review

**DOI:** 10.3389/fsurg.2018.00071

**Published:** 2018-11-27

**Authors:** Ferdinand Köckerling

**Affiliations:** Department of Surgery and Center for Minimally Invasive Surgery, Academic Teaching Hospital of Charité Medical School, Vivantes Hospital, Berlin, Germany

**Keywords:** incisional hernia, onlay technique, wound complications, surgical site infection, seroma, recurrence

## Abstract

**Introduction:** A meta-analysis that compared the onlay vs. sublay technique in open incisional hernia repair identified better outcomes for the sublay operation. Nonetheless, an Expert Consensus Guided by Systematic Review found the onlay mesh location useful in certain settings. Therefore, all studies on the onlay technique were once again collated and analyzed.

**Materials and Methods:** A systematic search of the available literature was performed in August 2018 using Medline, PubMed, Scopus, Embase, Springer Link, and the Cochrane Library. For the present analysis 42 publications were identified as relevant.

**Results:** In five prospective randomized trials and 17 observational studies the postoperative complication rates ranged between 5 and 76%, with a mean value of 33.5%. The recurrence rates in these studies also ranged between 0 and 32%, with a mean value of 9.9%. Hence, compared with the literature data on the sublay operation, more post-operative complications, in particular wound complications and seroma, with a comparable recurrence rate, were identified.

**Conclusion:** When the onlay technique is used in certain settings for incisional hernia repair, a careful dissection technique and prophylactic measures (drainage, abdominal binders, fibrin sealant) should be employed to prevent wound complications and seroma formation.

## Introduction

A meta-analysis that compared the onlay vs. sublay technique (1) in incisional hernia repair on the basis of two prospective randomized trials (RCTs) (2, 3), one prospective (4), and seven retrospective studies (5–11) identified significantly fewer surgical site infections and recurrences to the advantage of the sublay technique (1). Likewise, in a Danish registry study, the onlay technique was found to be a significant risk factor for a poorer long-term outcome (12). In one of the two RCTs, onlay mesh reconstruction in the large hernia group provided significantly better results than sublay repair (3). The recurrence rate was lower in the onlay group (12 vs. 20%; *p* < 0.05) (3). In an Expert Consensus Guided by Systematic Review the panel agreed that for open, elective ventral, and incisional hernia repair sublay mesh location is preferred, but onlay mesh location may be useful in certain settings (13).

This paper now once again critically analyzes the characteristics and findings of the available literature on the onlay technique in incisional hernia repair. To that effect, studies with a mixed patient collective comprising primary ventral hernias and incisional hernias had to be excluded due to significant differences in the outcomes (14–18). Particular attention should be paid to key questions, under which circumstances the onlay technique is advantageous and which factors are impacting the outcome of this technique.

## Materials and methods

**Figure 1:**
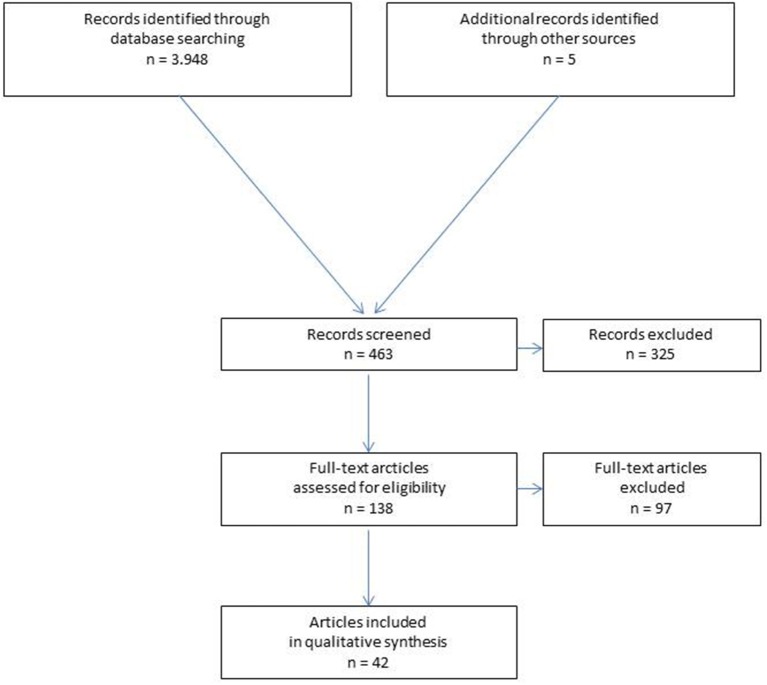
Prisma flow diagram of study inclusion.

A systematic search of the available literature was performed in August 2018 using Medline, PubMed, Scopus, Embase, Springer Link, and the Cochrane Library, as well as a search of relevant journals and reference lists. The following search terms were used: “Incisional hernia,” “incisional and ventral hernia,” “ventral hernia,” “hernia and onlay,” “ventral hernia and onlay.” The abstracts of 463 publications were screened (Figure 1).

For the present analysis, 42 publications were identified as relevant for the key question. According to the Prisma guidelines (19), a systematic presentation, and synthesis, of the characteristics and findings of the included studies has been made (Tables 1–3).

**Table 1:** Results of onlay technique with defect closure in incisional hernia repair in RCTs.

**References**	**Patients**	**Hernia type**	**Inclusion/Exclusion**	**Technique**	**Post-operative complications**	**Recurrence**	**Hospital stay**
Venclauskas et al. (2)	*n* = 57	Incisional	Male *n* = 22 female *n* = 35	Defect closure	Wound complications 49.1%, Seroma 45.6%	10.5% after 12 months follow-up	5.9 ± 2.3 days
Weber et al. (3)	*n* = 224	Incisional	Hernia orifice >25 cm2	Defect closure	— —	12% in a 5 years follow-up	— —
Natarajan et al. (20)	*n* = 13	Incisional	Exclusion of previous mesh repair	Defect closure	Seroma 38.5%, wound infection 16.7%	— —	— —
Sevinc et al. (21)	*n* = 50	Incisional	Exclusion of patients with a BMI >40	Defect closure	Wound complications 24%	6% with a median follow-up of 37.1 months	3.36 ± 1.9 days
Demetrashvili et al. (22)	*n* = 78	Incisional	Exclusion of recurrent incisional hernias	Defect closure	Wound complications 50%, Seroma 41%	5.1% in a mean follow-up of 4.6 ± 1.0 years	5.5 ± 2.7 days

**Table 2:** Results of onlay technique with defect closure in incisional hernia repair in observational studies.

**References**	**Patients**	**Hernia type**	**Inclusion/Exclusion**	**Technique**	**Post-operative complications**	**Recurrence**	**Hospital stay**
Kingsnorth et al. (23)	*n* = 95	Incisional	31% with recurrent incisional hernia	Defect closure	Post-operative complication rate 25%, Seroma rate 9.5%, wound infection 8.6%	3.4% after a median follow-up of 15.2 months	Mean length of stay 6.0 days (range: 2–44 days)
Andersen et al. (24)	*n* = 56	Incisional	Consecutive patients	Defect closure	Post-operative complication rate 13%	15% in a median observation time of 35 months (range: 4–151)	— —
Gleysteen et al. (6)	*n* = 75	Incisional	35% recurrent incisional hernias	Defect closure	Wound complication 21.3%, seroma 10.7%, wound infection 12.0%, hematoma 6.7%	20.0% after a median follow-up of 19 months	— —
Poelman et al. (25)	*n* = 101	Incisional	Minimum defect size 10 × 20 cm	Defect closure	Wound infection 21.0%, Seroma 27.0%	16% with a median follow-up of 64 months	4.5 days (quartiles 3-6.25)
Stoikes et al. (26)	*n* = 50	Incisional	4 patients with prior mesh procedure	Defect closure, mesh fibrin glue fixation	Wound complication 24% Seroma 16%,	0% after a mean follow-up of 19.5 months	Mean hospital stay 2.9 days (range: 0–15 days)
Alicuben et al. (27)	*n* = 22	Incisional	Clean, clean contaminated and contaminated cases included	Defect closure in 21 cases, bridging in 1 case, biological mesh	Wound complication 38.1%, Seroma 28.6%, wound infection 9.5%	4.8% (after bridge repair) in a median follow-up of 7 months (range: 2–14)	Median hospital stay: 7 days
Hopson et al. (28)	*n* = 20	Incisional	Defect size width or length ≥10 cm	Defect closure, Pro Grip Mesh	Wound complication 5%,	0% in 2 years follow-up	Same day *n* = 15, next day *n* = 5
Gemici et al. (29)	*n* = 154	Incisional	3 patient underwent additional abdominoplasty (1.9%) 7.1% urgent cases	Defect closure, full-thickness mesh fixation	Wound complication 43.7%, seroma 26.6%, wound infection 3.2%	5.2% in a median follow-up of 54 months (range: 12–96)	Mean hospital stay 4.9 days (range: 3–8)
Juvany et al. (30)	*n* = 76	Incisional	Exclusion of patients without 5-years follow-up	Defect closure	Wound complication 18.4%, seroma 10.5%, wound infection 2.6%	32% after 5-years follow-up	— —
Tuveri et al. (31)	*n* = 71	Incisional	Defect size >6 cm	Defect closure with incision of the anterior rectus sheath, biological mesh	Wound complication 76%, seroma 72%, skin necrosis 4%	1.4% in a mean follow-up of 40 months (range: 9–82 months)	Median hospital stay 6 days (range: 3–12)

**Table 3:** Results of onlay technique without or unknown defect closure in incisional hernia repair in observational studies.

**References**	**Patients**	**Hernia type**	**Inclusion/Exclusion**	**Technique**	**Post-operative complications**	**Recurrence**	**Hospital stay**
Kingsnorth et al. (9)	*n* = 16	Incisional	Lateral and transverse hernias	Defect closure unknown	Post-operative complication 31.2%	6% in a follow-up between 6 months and 6 years	Mean hospital stay 7.9 days (range: 6–50 days)
de Vries Reilingh et al. (10)	*n* = 13	Incisional	Large midline incisional hernias	No defect closure	Post-operative complication *n* = 17, seroma 69.2%, wound infection 23.1% skin necrosis 23.1%	23.1% in a median observation time of 19.4 months	— —
Machairas et al. (32)	*n* = 43	Incisional	56% recurrent incisional hernias	No defect closure	Wound complication 21%, seroma 14%, wound infection 7%	9.3% in a mean follow-up of 54.4 months (range: 4–106 months)	6–8 days
Coskun et al. (11)	*n* = 22	Incisional	— —	Defect closure unknown	Wound complication 22.7%, seroma 4.5%, wound infection 9.1%	13.6%	7.9 days (range: 5–11 days)
Abdollahi et al. (8)	*n* = 33	Incisional	Emergency cases excluded	Defect closure unknown	Wound complication 9.1%	6.1% in a mean follow-up of 98 months (range: 48–174 months)	— —
Kumar et al. (4)	*n* = 45	Incisional	— —	Defect closure unknown	Wound complication 37.8%, seroma 24.44%, wound infection 13.33%	10.8% in a follow-up of 2–24 months	— —
Memon et al. (33)	*n* = 60	Incisional	Defect size ≥10 cm	Defect closure unknown	Surgical site infection 21.7%	6.7% in a mean follow-up of 20.05 months (range: 12–48 months)	— —

## Results

### Incisional hernia repair in onlay technique in meta-analyses

A meta-analysis of the Cochrane Library (34) that included two RCTs reporting on only incisional hernias (35, 36) did not identify any significant difference between the open onlay and the intraperitoneal onlay mesh (open IPOM) technique.

The meta-analysis by Timmermanns et al. (1) that included two RCTs, one prospective and seven retrospective studies (2–11) with 775 onlay operations and 1,173 sublay operations in incisional hernia repair observed a trend for recurrence in favor of sublay repair (odds ratio = 2.41; 95% CI 0.99–5.88; *p* = 0.05). Surgical site infection occurred significantly less often after sublay repair (odds ratio 2.42; 95% CI 1.02–5.74; *p* = 0.05).

No difference was observed regarding seroma and hematoma (1).

### Results of onlay technique in incisional hernia repair in RCTs

In the meantime, the findings of five RCTs reporting on the use of the onlay technique in incisional hernia repair are available (Table 1) (2, 3, 20–22). In all RCTs, defect closure was carried out as part of the onlay technique. The wound complication rate for the onlay technique in all RCTs was between 24 and 49.1%. The most common wound complication was seroma formation as seen in between 38.5 and 45.6% of cases. Conversely, the recurrence rates were within an acceptable range of between 5.1 and 12% at follow-up of 1–5 years.

### Results of onlay technique for incisional hernia repair in registries and multicenter observational studies

In a nationwide prospective study of the Danish Ventral Hernia Database conducted between January 1, 2007 and December 31, 2010, 454 from 3,258 incisional hernias were repaired with onlay technique (12). The cumulative risk of recurrence repair after open and laparoscopic repair was 21.1 and 15.5%, respectively (*p* = 0.03). Younger age, open repair, hernia defects >7 cm, and onlay mesh positioning in open repair were significant risk factors for poor late outcomes (*p* < 0.05) (12).

In a Swedish study reporting 869 incisional hernia repairs from 40 hospitals the recurrence rate for the onlay technique 12–24 months after surgery was 19.3% (5).

In a retrospective study of 16 Veterans Affairs Hospitals, 1,346 elective incisional hernia repairs, of which 30% in onlay mesh technique, were analyzed (37).

Compared with suture repair, the onlay mesh technique did not substantially reduce the recurrence risk (37).

### Results of onlay technique for incisional hernia repair with defect closure in observational studies

The findings of 10 observational studies (6, 23–31), which describe defect closure as part of the repair technique, are available on onlay incisional hernia repair. The wound complication rates were reported to be between 5 and 76%, the seroma rates between 9.5 and 72% and the recurrence rates between 0 and 20.0% (Table 2). In the study by Tuveri et al. (31) with a very high wound complication rate of 76% and a seroma rate of 72%, defect closure involved incision of the anterior rectus sheath and the use of a biological mesh. The lowest post-operative complication rate with 5% and a recurrence rate of 0% in a follow-up of 2 years was published by Hopson et al. (28) in incisional hernias with a defect size in width or length not larger than 10 cm and the use of a self-fixating mesh. A lower post-operative complication rate of 13% and a recurrence rate of 15% in a median follow-up time of 35 months (range 4–151 months) was also reported by Anderson et al. (24) in a consecutive series operated by 4 senior surgeons of a single institution.

### Results of onlay technique for incisional hernia repair without or with unknown defect closure

In seven other observational studies (4, 8–11, 32, 33) on the onlay technique in incisional hernia repair, defect closure was not performed or whether defect closure was carried out was not described as part of the surgical technique. In these studies, the wound complication rate was reported to be between 9.1 and 37.8%, seroma rate between 4.5 and 69.2% and the recurrence rate between 6.1 and 23.1%.

The lowest post-operative complication and recurrence rate in this subgroup was demonstrated in a case series of 354 incisional hernias with a very selected indication for onlay repair (8).

### Mean values of post-operative complication and recurrence rates

Overall, on evaluating the results of all studies together the mean value for the post-operative complication rate was 33.5%, with a range from 5 to 76%, and for the recurrence rate it was 9.9%, with a range from 0 to 32%.

## Discussion

In the meta-analysis by Timmermanns et al. (1) comparing sublay vs. onlay incisional hernia repair, fewer surgical site infections as well as recurrences were identified in favor of the sublay technique. That was also confirmed by data from the Danish Hernia Registry (12). Nonetheless, an Expert Consensus Guided by Systematic Review found that, while the sublay operation should be given preference for incisional hernia repair, the onlay mesh location might be useful in certain settings (13). Therefore, in this present review the available data on the onlay technique in incisional hernia repair were collated in order to compare this method with the sublay technique (38). In this analysis, too, it was revealed that the onlay technique was associated with a higher post-operative complication rate, with a mean value of 33.5% and range from 5 to 76%, than the sublay technique, with mean value of 18.6% and range from 8 to 26% (38). The mean value for the recurrence rate in the onlay technique was 9.9% with a range from 0 to 32% and, as such, was comparable with the results of the sublay operation with mean value of 13.5% and range from 1.6 to 32% (38). Hence, the main difference between the sublay and the onlay technique was a higher post-operative complication rate to the disadvantage of the onlay technique. Since these complications were generally wound complications and seroma it is thought that they were attributable to the more extensive dissection in the abdominal wall for exposure of the anterior rectus sheath and the anterior abdominal wall fascia for mesh placement in the onlay position (1). Surgical experience, selective indications, and smaller defects seem to reduce the post-operative complication rate (8, 24, 28). Whether continuous drainage of the wound area in the onlay technique could improve the outcomes cannot be ascertained at present on the basis of the existing literature (39, 40). Therefore, the role of drains in open incisional hernia repair should be investigated in further studies (39, 40). Additional preventive measures against post-operative seroma formation in open incisional hernia repair could entail wearing abdominal binders for several weeks and/or the use of low-thrombin fibrin sealant (41, 42).

Therefore, future studies on the onlay technique in incisional hernia repair should involve selected indications, a standardized surgical technique by experienced surgeons, paying special attention to ensuring careful dissection in the abdominal wall and to the incorporation of preventative measures against seroma formation.

With regard to the recurrence rate, the onlay technique appears by all means to be comparable with the sublay operation. If the onlay technique outcomes can be improved through technical standardization and the consistent use of measures aimed at reducing the seroma rate, according to the Expert Consensus (13) the onlay technique could indeed be useful in certain settings. Therefore, the onlay technique, should be further investigated in good studies in the future, while focusing in particular on identification of the settings in which the onlay technique has advantages over other surgical procedures.

In conclusion, it must be stated that based on the available literature the onlay compared with the sublay technique in incisional hernia repair is associated with markedly more wound complications and seroma rates and with a comparable recurrence rate. Therefore, in the onlay technique the occurrence of wound complications and seroma formation must be prevented through selective indications, surgical experience, careful dissection in the abdominal wall, and prophylactic measures such as drainage, abdominal binders, fibrin sealant. Furthermore, those settings in which the onlay technique has advantages must be better defined.

## Author contributions

FK: literature search, design of study, concept of manuscript, and final submission.

### Conflict of interest statement

The author declares that the research was conducted in the absence of any commercial or financial relationships that could be construed as a potential conflict of interest.
